# The Nursing Supplement

**Published:** 1888-09-08

**Authors:** 


### The Nursing Mirror.

All Contributions for this Supplement should be addressed "The Nursing Mirror," care of The Editor, 38, Old Jewry, E.C.

### En Passant.

HOLIDAY HOUSE.—During the summer months there is a country house not far from Edinburgh, which opens its doors to the poor children of that city. Every fortnight twenty little ones go down to Holiday House, and twenty little ones laden with flowers and bright memories return to Edinburgh. The matron of the establishment, Miss Aherne, has her work cut out to care for this large family, and as there are often sickly ones amongst them, she has a tiny bedroom adjoining her own, as a sort of sick ward. It must be quite as tiring to be "nurse" to twenty healthy children, as "sick-nurse" to fewer feeble ones.

PERSONAL ITEMS.—The many friends of Mrs. Webb, the late matron of the Lewes Dispensary and Infirmary, will regret to hear that she is lying dangerously ill at the house of Mr. Luckin, in South Street, where she removed after leaving the institution. Little hope is given of her recovery. Miss Steuart, late sister-superintendent of the Middlesex Institute, has been dangerously ill with diphtheria, at the Fever Hospital. We are glad to hear she is getting better. It is rumoured that Miss Lloyd will resign her post as Mother Superior of the Nursing Sisters of St. John-the-Divine this autumn, and that Sister Selina may very probably take her place. Miss Baylis (who, we learn, is one of Mr. Wilson's nurses) progresses well.

KNOWLEDGE FOR NURSES.—The nurses' sitting-room at the London Hospital received a most welcome addition this summer, in the shape of a cupboard full of beautifully prepared specimens presented by Frederick Treves. The question as to how much a nurse ought to know is one which constantly occupies the medical mind, and whereas some doctors think that the greater the knowledge the better the nurse, many others prefer that ignorance which is supposed to be the parent of obedience. That a well-known surgeon like Mr. Treves should present such a gift to the hospital of which he is surgeon shows that greater trust is being placed in the nurses of the present day, and that every incentive is being given to encourage them to thoroughly master their profession. The specimens were prepared by Mr. Openshaw, the curator of the Medical School Museum, and include those internal organs of which it is absolutely necessary every trained nurse should have some knowledge.

THE NEW YORK CLIMATE.—An American correspondent writes to us about the description of the climate of New York, given in an article on the Bellevue Hospital in our issue of June 30th: "There are some days in summer that are comfortable in the city, and there have been many this year, but to a stranger they would seem intolerable unless he had had some experience of a tropical climate. Our richer class go to summer resorts and to California and Europe, the poorer visit their friends on farms or in the suburbs. It is the poor and the working people, the latter including professional men, men of business, clerks, type writers, women in all kinds of employment (such as nurses) who suffer. But they have a respite in the way of cool waves which may last two days, a night, or perhaps in exceptional cases, a week. Once in a while, we get caught by a hot wave; for instance, when I last wrote you, the latter part of June, it should have been the most delightful season of the year, fresh and cool as May. A hot wave came from the south, and took possession of the city when people were unprepared for it. We were in the midst of our summer shopping and packing, the thermometer ran up to 104 deg. in the shade (Farh.), and the result was that many people were prostrated, this wave lasted for ten days, by the end of that time New York was deserted."

A SUGGESTION FOR SISTERS.—In all general hospitals there are often patients who, after the first crisis of an illness or operation, receive but little attention from the staff, and are lelt chiefly in the nurse's hands. Unexpected deaths among these semi-convalescents or chronics, who have dragged along for months, are not uncommon. The Sister then, should pay special attention to these uninteresting patients, keep them under close observation, and on noticing any change of symptoms, ask for instructions. The press of more serious cases should never cause these wearisome cases to be neglected, or they may never emerge from their dangerous obscurity till their sudden death is a shock to all.

NURSES ON THE CONTINENT.—At last Mrs. Brewer's interesting articles in the *Sunday at Home* have reached the point looked for by London nurses, and begun to describe nursing work on the Continent. In the chapter in this month's magazine the three hospitals at Dresden, which are under the charge of the sisters of the Albert Verein, are described. These are the Carola House, the Convalescent House, and the Hospital for Little Children. Mrs. Brewer's opinion evidently is that nurses are overworked in Dresden, and that the training is very hard. Some two months ago we gave an account of the Albertinerins, and described their course of training as not only thorough, but often finishing. Mrs. Brewer had the honour of an interview with the Queen of Saxony, who founded this Order, and who is deeply interested in nursing. We hope Mrs. Brewer said a word in favour of shorter hours and lighter duties for the poor, overworked nurses. The sisters attend entirely to the dispensary, and make up all the prescriptions; they also have to learn to cook. We look forward eagerly to further papers by Mrs. Brewer.

THE POLICE COURTS AGAIN.—Two more nurses have been making their appearance in the police-courts, and both on the same day. The first was a nurse at the Victoria Children's Hospital, who was charged with assaulting the mother of a small patient. The complainant said she was a widow, and on Saturday, the 18th ult., in the evening she called at the hospital to ascertain the last words of her little daughter Ethel, aged five and a-half, who died there on June 11th. She told the nurse (the defendant), who saw her in the vestibule, that she only had notice of her daughter's death on "a dirty halfpenny post-card," and she considered that a lady of her position should have been telegraphed to several times a-day. The defendant took hold of her and threw her out in the roadway, causing her to fall heavily. She thought she must have been kicked while she was down, and she was very much bruised and hurt. Upon cross-examination, however, a most miserable story was elicited from the woman, who admitted that she had never been near her daughter for months. The charge was absolutely unfounded, and the magistrate dismissed the summons, with costs. The second case was that of a well-dressed woman, evidently much distressed in mind, who applied to Mr. Biron to obtain protection from her husband from whom she said she had been separated fourteen years. She had consequently been compelled to get her own living, and was engaged as a nurse, and at the present time was attending a lady. Her husband had found her out, and daily persecuted her for money. He kept ringing and knocking at the house, disturbing the patient, and she was frightened that she would be turned away in consequence. She had been obliged to leave other places by reason of his annoyance. Evidently a nurse's life is not exempt from external trials and troubles.

### Original Articles.

### SICK NURSING AMONG THE POOR.—AN AMATEUR'S VIEW.

In entering upon this subject, we should like first to record our belief that in no rank of life is there more genuine, unostentatious self-sacrifice and unselfishness shown in times of sickness than among the very poor. It is true that we occasionally meet with hideous instances of unnatural neglect and brutal treatment, and no doubt there is much lack of wisdom, tact, and intelligence in the ministrations of the best-intentioned; but we believe that all who have really worked among the poor, either in town or country, will concur in the assertion that we have made. How often one has been put to shame by the brave (if plaintive) determination of the][delicate mother, with a young baby to attend to, to "keep about" because an ailing child or sick husband must be placed first in the list of invalids. How often one hears, casually, of a hard-working woman, whose labour power by day means a large proportion—sometimes the whole —of the support of her family, giving up her night's rest to attend upon a sick neighbour.

We, in happier circumstances, who consider ourselves entitled to a day of rest and dawdling after a night of sick nursing, can scarcely realise what it must be to sacrifice sleep, that precious boon to the hard worker; "sore labour's bath —chief nourisher in life's feast"—with the prospect of unslacked toil upon the morrow.

But with all this self-devotion, what a curious inattention is shown to the feelings of the patient, and, at times, what an extraordinary callousness (as it seems to us) at the prospect of losing a loved one. No doubt, the *penchant* for sensationalism which prevails among the poor renders a certain fuss and excitement, which would be unendurable to a refined invalid, rather grateful to the feelings of a cottage patient. We have known instances of sick persons listening with great apparent complacency while their attendant detailed to the visitor how she never thought they could have lived through the night. Some women will enumerate in a tone almost of triumph the children or other relatives whom they have "buried," often in the hearing of an invalid whom they opine will be "just like the rest."

In cases of such want of reticence we console ourselves with the hope that the talkers know how much they themselves would tolerate or like under the same circumstances, and that they understand the patient's frame of mind better than we do. But acquiescence is not so easy when we come face to face with some of the extraordinary sanitary and medical notions of the poor. True, the complaints they suffer from are often removed from the ordinary medical *répertoire*, and special treatment may be required for such disorders as "the hairy siplus," and "the bran titus," or, as another version of that long-suffering bronchial affection hath it, "the brown typhus." We have heard of a case of the latter when the doctor was reported to have "seen the *brown* coming out all over."

"Physics in the neck" seems, at first hearing, to indicate merely a passing awkwardness on the part of the nurse; but we find, on further inquiry, that it represents the diagnosis of the actual complaint of the sufferer. There are many persons in all ranks, we imagine, who would be ready to confess to having suffered from "the concertina;" but most of these would be referring to the instrument so designated, and not to the virulent disease for which the name has also been found to do duty.

In some circles, to have a great deal of information, is considered very satisfactory and beneficial; but it is possible, we find, to have "information of the lungs" in a style which is very far from pleasing.

Such being their typical disorders, it is not, perhaps, wonderful that many of the remedies in vogue among them should be somewhat peculiar. Both "turcomtine" and "camphire" are included in their pharmacopeia, and, no doubt, are exceedingly useful; but the rubbing of salt into a burn "to bring out the fire," does not recommend itself to us, although it may have some dim connexion with the use of salt when a chimney is burning. We should be glad to be fortified with high medical authority before venturing to pronounce an opinion upon the following practice: "My 'usband was bad with the gout, so I inoccle-lated 'im with a bee— stung 'im with a bee in the foot."

Preventive measures are not, as a rule, popular among the poor, but we have met with a stalwart female (of the species sometimes called "a resolute pardner") who announced it to be her practice to administer to her meek spouse "a spoonful of Friar's Balsam every night of his life." We have not yet ascertained what disease this treatment was supposed to ward off from the very patient patient.

And that word "patient," by the way, must not be considered to apply too stringently to those only who are suffering from illness. We were, on one occasion, puzzled to hear continued reference made to "the doctor's patient," as one who had recommended such-and-such remedies. In this case the "patient" was evidently not the invalid, but belonged, it seemed, to that class of verbs which "do" rather than "suffer." The mystery was explained at last—the title of "doctor's patient" had been bestowed upon the medical man's "assistant."

But, passing from these absurdities, it is certainly sad to encounter the utter want of common sense in sanitary matters which prevails even among practical and, in other respects, sensible cottage women. Cold is the great dread, and an idea prevails that fresh air is unattainable without it; hence windows are kept carefully and tightly closed, and a state of atmosphere is maintained which, from closeness and unsavouriness is almost unendurable to luckless visitors. The notions as to invalid diet, too, are often such as to irritate the calmest tempered medical attendant. To find a patient, just beginning to recover from typhoid fever, for instance, being indulged, in his restless longing for solid food, with a nice little bit of cold pork and potato, or a bit of pastry and some warm beer, must be disheai tening in the extreme.

Cottage hospitals do their best to remedy these evils, and we trust that, by degrees, the foolish prejudice existing against them among the poor may be overcome.

But we think that the greatest boon of all is conferred by those nursing sisterhoods, whose members go into the very homes of the sick poor, and show what may be done with the simple appliances at hand, and how comfort and health may be studied without any costly apparatus, but merely by the use of that nursing instinct which exists in most women, guided by the laws of common sense. Terra Cotta.

### A CRIPPLES' RACE.

"A sister remembers still (with a shudder) what were her feelings when, having turned away for a few moments to attend to some other children, she heard terrible sounds of screaming a little way off. Hurrying to the spot from whence the cries proceeded, she saw three cripples prostrate on the sand, all crying lustily, their crutches lying beside them forlornly. Several other lame children were limping along not far off, all making for the same spot. When tears were wiped away and it had been ascertained that no one was hurt, Sister A—— asked how the accident had happened. 'We was having a cripples' race,' said the children; 'and it was great fun till we tumbled down!' The cripples always are the most spirited and enterprising little folks, yet certainly a race on crutches was *not* what one would have expected or encouraged."—*Our Work.*

### Letters from a Probationer.—II.

Dear ———, I am sitting in Sister's little room while I write this, from which you will guess that I am ready to sub scribe to the truth that first impressions are not always correct. I have got to like Sister very much, and to make every allowance for her if she is sometimes sharp. I haven't been very well these last two days, and she has been so good to me, and has now sent me into her own little sanctum for half-an-hour's quiet. The window at which I am sitting is wide open, and looks down on to the busy street where the traffic is roaring by. It is visiting-day, and a large crowd is already congregating outside the great iron gates, though they will not be admitted for twenty minutes yet. The patients are only allowed to receive their friends twice a-week, unless they are dangerously ill; and the two visiting afternoons are always eagerly looked forward to. I can see a stout, motherly old woman sitting on the kerb, who, I am sure, must be the wife of the old farmer who has come to us to be cured of obstinate sciatica. She has a huge bunch of roses, geraniums, snapdragon, nasturtiums, and old-fashioned flowers, all as highly-coloured as her own florid cheeks. Her three-cornered shawl and black silk bonnet look so respectable compared with the garb of most of those who are waiting. Our general style of visitor is an under-sized girl with a huge fringe and dirty face. Two long earrings of brass dangle in her ears, her large hat is trimmed with soiled feathers; round her neck is a faded blue hand-kerchief, and her skirt is composed of numerous small flounces. Sometimes she is loud and impudent, and at first I simply detested to look at her, but now I know her better I pity her with all my heart. One poor patient, who is partially paralysed and afflicted with bed sores, and who is slowly dying, has a most faithful friend in just such a girl. She comes every evening as soon as her work is over and sits beside him till ten or eleven, and once or twice lately she has stayed all night. As she passes to her work in the early morning she always runs in to see how he is, and her patience with him is remarkable, for he is a trying man to deal with. One of our great difficulties is to prevent friends from bringing in unsuitable food to the patients. We had a great row last Sunday, and I was terribly mortified; one visitor had smuggled a flask of gin in to one of our patients, and towards evening the man got excited with drink and became abusive to the patient in the next bed, finally springing up and attacking him. Sister sent me for the house physician, who immediately divined the cause of mischief and searched the bed: he found the empty flask. The man was not very ill, and always very trouble-some and ungrateful; so the house physician determined to make an example of him and discharge him on the spot. The house governor was sent for to give his authority to the proceedings, and the man was then dressed and carried down to a cab by two porters. The house governor stayed behind, however, to deliver a little lecture to sister and nurses on their carelessness in permitting the spirit to be smuggled in. So you see visiting day is not all joy to us, though the patients appreciate it. We like the flowers the visitors often bring the nurses, though they are constantly the refuse of Covent Garden, and so old and stalkless that they wither the same night. There are two men with barrows busily selling such flowers outside the gates just now, and what is worse, there is a barrow of unwholesome highly-coloured sweetmeats, and a stall of cheap fruit, either unripe or rotten, all of which are temptations to the visitors to break the rule which forbids them to bring food to their friends. There! the gates are thrown open just as the clock strikes, and the crowd comes streaming in, each person holding up the blue-ticket which secures them entrance. I can hear the hurrying footsteps along the stone passages, and see the eager eyes of the patients fixed on the door. I must return to the ward and watch, that last Sunday's scene may not be repeated again.

### Notes from Australia.

(*By Our Own Correspondent.*)

"At Home," *i.e.*, in England, you have but a faint idea of the size, energy, and culture of this great city of Melbourne. There are Irish Radicals even here, but on the whole there is a striking love and enthusiasm for the mother country and her institutions, and a rousing desire to follow in some of the good old ways, as well as in some of the good new ones Nursing and the instructions given by the St. John's Ambulance Corps have a no mean place in the care of the leaders of benevolent work. Hospitals are becoming numerous, and are, we believe, well and efficiently managed. That the old and the new system of nursing are both in use is not surprising. At the Melbourne Hospital, nursing is managed in the old style,* the nurses going through the various stages of promotion according to merit; they all begin as scrubbers, and are chosen in the first instance from among women who are attracted by a perennial daily advertisement in the papers, "Woman wanted as assistant nurse; Melbourne Hospital." In the Alfred Hospital, in Melbourne, and the Prince Alfred Hospital, New South Wales, the more modern style prevails, the nurses entering as probationers, and receiving a certain amount of tuition.

Some doctors, while they admit that they cannot say which of the ways is better, say they have a strong leaning to the Melbourne Hospital system, and further, that as yet it has produced much better nurses; but the reason for this may be not so much in the system as in the fact that hitherto the number of trained lady nurses in Melbourne has been very small. Meanwhile, there are people who say that more patients die in the Melbourne Hospital, in proportion to their numbers, than die in the other hospitals.

Some particulars about the Homœopathic Hospital, the Children's Hospital, and the Convalescent Home we will give in time, as well as some details about the Immigrants' Home, the Sailors' Home, the Orphanage, and the Blind Asylum. Unfortunately, the latter is a great need in the colony, as the heat is dangerous to eyesight, and often people do not take early enough precautions to save their sight.

Dr. R. Schlesinger has been giving a course of lectures this summer to the pupils in training and the nursing staff of the Alfred Hospital. There was a large attendance of nurses and lady visitors.

Dr. Seelenmeyer is now giving a course of lectures on anatomy, to nurses and ladies at the Homœopathic Hospital.

Mrs. Turnbull is president of the Women's Hospital. This hospital takes two classes of pupil nurses: the first pay a fee of twelve guineas and serve for six months; the second pay a fee of one guinea and serve for twelve months. The report of the Women's Hospital, for the week ending July 19th, was as follows: 19 patients remained in the hospital on the 12th inst., six were admitted during the week, 11 were boarded out, 12 discharged, and 13 remained; total number of out-patients for the week, 125. This gives some idea of the experience in nursing to be gained there.

Melbourne, July 28th.

* They have quite recently begun to take pupils for training as nurses.

### Exchange and Mart.

Under this heading we propose to insert purely Exchange advertisements, at a charge of sixpence for eighteen words, or three insertions for one shilling It must be distinctly understood that ordinary advertisements will not be inserted here.

WANTED.—"Quain's Dictionary," in fair condition. Would give a pocket spectroscope, which cost twenty-five shillings. Address Student, Office of The Hospital.

APRONS.—Half a-dozen white linen aprons with bibs. Will exchange for nurse's surgical case. Nurse Norma, Office of The Hospital, 38, Old Jewry, E.C.

### Everybody's Opinion.

[*Correspondence on all subjects is invited. No communications can be entertained if the name and address of the correspondent is not given, or unless one side of the paper only be written on.*]

### THE ZENANA MISSION.

YOU were kind enough to insert my letter about the College in your interesting paper. I therefore venture to send two extracts from letters since received from ladies who were trained at the College, both of which show how valuable they have found the training.

Benares, India, July 20th.—Mrs. Tresham writes: I was glad to see that Dr. Griffith had had a good annual meeting for our Home; but, oh! my heart just ached that I could give no help to the Irish and Syrian ladies. I do not consider it generous of some ladies who have talents, time, and money to make such disparaging remarks of those with fewer talents. Such would be well answered through a little patient of ours. Some while ago its parents thought the little fellow would scarcely live, yet God has raised it up through you, my dear Mrs. Rumford. Every day of my life have I had reason to be thankful for the useful knowledge I gained under Dr. Griffith and our lecturers. Often the only result we know of is a fresh applicant for the same medicine as "a neighbour had, and is cured." Is it not even more convincing when the proof comes from an infant's face. On the first day we did not see him open his eyes, but yesterday and previously he smiles and crows. These parents have lost their children till this one, and their happy faces and confidence are worth having.

Mission School, Schwifat, Beyrout, Syria, July 23rd.—Dear Dr. Griffith—I was glad to read the account of your annual meeting, and to find you were making such good progress in every way. Whatever objections are now being made to a partial medical training, I, for one, can bear my thankful testimony to the value of the training I received at the Zenana Medical College—the only place where I could have obtained it in the time I had to spare. When working out here, before going there, I saw many suffering, and knew not what to do for them; now, with the blessing of God on the knowledge obtained at the College, I have been enabled to relieve many. Here, where there is no resident medical man, they are thankful to come to my dispensary, which is open twice a-week. Three hundred and seventy-five patients have been prescribed for since the beginning of the year—mostly the ordinary complaints of women and children, though I have had other cases. One, a little child of seven, who had been frightfully bitten by a dog, cost me some anxiety. I remembered the instructions I had heard at the College, and, following them, found the treatment was successful, and the child's health has not suffered. My anatomy class are continuing their studies, and received much praise for their answers this examination. I long to know if your committee will be able to admit two of them as free students at the College; I have been anxiously watching for an answer to my letter on that subject. My hope is to have a hospital here for women and children, and I would desire such a training for them as to enable them to take the superintendence of it. They belong to the leading families, and so, if trained, would be suitable for such a post.—Your grateful pupil, L. PROCTOR. MARY A. ROBINSON.

### THE QUESTION OF COURAGE.

IN answer to article on "Courage" in the "Nursing Mirror" of THE HOSPITAL, on August 25th, I beg to state that a nurse who is nursing small-pox runs no risk if revaccinated. Many people run away with the idea that in nursing small-pox a nurse must be most courageous. There is a certain amount of delirium in small-pox, and some are unsightly. As it is impossible for a nurse who has been re-vaccinated successfully to take small-pox, I do not think they are so courageous as people think. I think the nurse who nurses typhus, typhoid, or scarlet fever, or any kind of infectious disease, the most courageous, as she is in constant danger of failing with any of the above-named diseases.

M.

### Appointments.

[It is requested that successful candidates will send a copy of their applications and testimonials, with date of election, to THE EDITOR, The Lodge, Porchester Square, W.]

MISS HILLIER has been appointed sister superintendent of the Middlesex Hospital Trained Nurses' Institute. Miss Hillier trained at the Middlesex two years ago as a lady probationer, and since then has been sister at the Brompton Hospital for Consumption, and at the Hospital for Women, Brompton.

BRADFORD INFIRMARY.—Miss Rose Dickenson has been appointed lady superintendent of this infirmary. Miss Dickenson is a St. Bartholomew's nurse, and for some time has held the post of Sister John. Her departure from Barts. will be regretted by many friends.

CARLISLE FEVER HOSPITAL.—Miss M. Harris has secured the post of matron at this hospital. Miss Harris was trained at Addenbrooke's, Cambridge, during the matronship of the late Miss Alice Fisher, and accompanied her to the Radcliffe Infirmary. For the last two years Miss Harris has had charge of a ward at the Leeds Fever Hospital, so that she is thoroughly fitted for the post she has now attained.

### Vacancies.

The following vacancies are announced:—

**Matrons.**

Bedford General Infirmary; £50.
Victoria Home for Nurses, Bournemouth.

**Night Superintendent.**

Monsall Fever Hospital; £40.

**Nurses.**

Barnhill Hospital, Glasgow; £24.

Bradford Eye and Ear Hospital; £25.

Sheffield Nurses' Home; £25.

Dispensary Hospital, Bury; £25.

Homes for Aged Mariners, Egremont £25.

Kent Nursing Institution.

Brighton Borough Fever Hospital; £25.

Kingston Nurses' Home, Baker Street, Hull; several.

Hospital for Epilepsy and Paralysis; £20.

Carrickfergus Nursing Society.

Bristol Infirmary; £25.

Southport Nurses' Home..

Northampton Institute.

Canterbury Institute.

Radcliffe Infirmary.

Miss John's Home, Glasgow.

Brighton and Hove Hospital for Women; £30.

Devonport Nursing Institute; £25.

Irvine Poorhouse; £25.

London and Brighton Nurses' Institute; several.

Tunbridge Wells Nurses' Institute; several.

**Probationers.**

Royal Hospital for Diseases of the Chest.

Batley Cottage Hospital, Yorkshire.

Lowestoft Hospital.

Canterbury Institute.

Kent and Canterbury Hospital.

Lloyd Cottage Hospital, Bridlington, E. Yorks.

**District Nurse.**

Ayr Sick Poor Nursing Association; £50.

**Sister.**

Borough Fever Hospital, Leeds; £30.

**Ward Maids.**

Victoria Hospital, Burnley; two; £12.

### Notes and Queries.

TO CORRESPONDENTS.—1. Questions may be written on post-cards. 2. Advertisements in disguise are inadmissible. 3. In answering a query please quote the number. 4. If a private answer is desired, a stamped addressed envelope must be forwarded.

(50) *A General Certificate.*—Will anyone be so kind as to inform me which of the London hospitals would be the best for me to apply to? I have had two years' medical training, but am anxious to get a good general certificate, where the work is not very rough. Is King's College one of the best, and is it very difficult to get a vacancy there? Most of the hospitals seem to have so many waiting, that is almost hopeless unless one pays. Is there any hospital except the London where the training is for less than three years? I shall be most grateful to anyone who will kindly tell me this. —NURSE MARY.

(51) Can you inform me of any name and address of a good general hospital in Brisbane, Queensland, Australia? If not, would you kindly insert inquiry in your next issue of THE HOSPITAL?—NURSE FARRAR.

(54) Nurse D. will be glad to know where she can receive a thorough training in massage and electricity at most moderate terms.

**Answers.**

(50) *Nurse M.*—Refer to an article in the *Sunday at Home* for July. At the Middlesex, University College, and Charing Cross Hospitals, probationers paying a fee of one guinea a-week are exempted from a large amount of ward-work, such as dusting, the cleaning of tins, brasses, &c. They are not bound after their year of training. Courses of lectures are given at the first three hospitals, and at the Middlesex.

(52) *Convalescent Hospital.*—Would E. D., whose query with regard to a convalescent home for children appeared in our issue of August 11th, please send her address to Miss Jones, Walden House, Worcester Road, Sutton, Surrey?

(53) *A Reader Thereof.*—We certainly did not cull the story from the coloured pages of which you are a reader. It was told us by a military man from India a very long time ago.

